# Intracellular Degradation of SARS-CoV-2 N-Protein Caused by Modular Nanotransporters Containing Anti-N-Protein Monobody and a Sequence That Recruits the Keap1 E3 Ligase

**DOI:** 10.3390/pharmaceutics16010004

**Published:** 2023-12-19

**Authors:** Yuri V. Khramtsov, Alexey V. Ulasov, Tatiana N. Lupanova, Tatiana A. Slastnikova, Andrey A. Rosenkranz, Egor S. Bunin, Georgii P. Georgiev, Alexander S. Sobolev

**Affiliations:** 1 Laboratory of Molecular Genetics of Intracellular Transport, Institute of Gene Biology of Russian Academy of Sciences, 34/5 Vavilov St., 119334 Moscow, Russia; ykhram2000@mail.ru (Y.V.K.); ulasoff@yandex.ru (A.V.U.); tatyanalupanova@yandex.ru (T.N.L.); tslast@genebiology.ru (T.A.S.); aar@genebiology.ru (A.A.R.); bunin.e.001@gmail.com (E.S.B.); georgiev@genebiology.ru (G.P.G.); 2 Faculty of Biology, Lomonosov Moscow State University, 1-12 Leninskie Gory St., 119234 Moscow, Russia

**Keywords:** modular nanotransporters, Keap1E3 ligase, N-protein degradation, SARS-CoV-2 virus, flow cytometry, western blot, intracellular concentrations, thermophoresis, cathepsin B

## Abstract

The proper viral assembly relies on both nucleic acids and structural viral proteins. Thus a biologically active agent that provides the degradation of one of these key proteins and/or destroys the viral factory could suppress viral replication efficiently. The nucleocapsid protein (N-protein) is a key protein for the SARS-CoV-2 virus. As a bioactive agent, we offer a modular nanotransporter (MNT) developed by us, which, in addition to an antibody mimetic to the N-protein, contains an amino acid sequence for the attraction of the Keap1 E3 ubiquitin ligase. This should lead to the subsequent degradation of the N-protein. We have shown that the functional properties of modules within the MNT permit its internalization into target cells, endosome escape into the cytosol, and binding to the N-protein. Using flow cytometry and western blotting, we demonstrated significant degradation of N-protein when A549 and A431 cells transfected with a plasmid coding for N-protein were incubated with the developed MNTs. The proposed MNTs open up a new approach for the treatment of viral diseases.

## 1. Introduction

The importance of new antiviral drug development has been especially reinforced by the SARS-CoV-2 pandemic. Targeting and inhibiting the activity of key viral proteins involved in SARS-CoV-2 replication and survival is a matter of intense continuous research due to the virus’s adaptive capacity, leading to the emergence of new strains and variants. Coronavirus nucleocapsid protein (N-protein) is an attractive viral target, contributing to the different aspects of virus biology: replication, viral assembly, budding, immune system interference, and determinants of virulence and pathogenesis [1,2,3,4]. Moreover, the N-protein of SARS-CoV-2 is highly conserved (90% sequence homology with that of SARS-CoV-1) and has fewer mutations than the spike protein [2,3]. Several small molecules are currently under active investigation, exploiting N-protein targeting (reviewed in [2,3]). In this paper, we suggest an alternative way to inhibit SARS-CoV-2 N-protein, exploring recently emerged direct protein degradation technologies, recruiting cellular protein control mechanisms such as ubiquitin–proteasome and autophagy systems [5,6], which are in many respects better suited for protein targeting. Compared to small-molecule approaches, the protein degradation strategy offers a number of strengths: direct elimination of proteins, all target protein-associated functions attenuation by one shot, substoichiometric activity due to the catalytic mechanism of action, and long-lasting inhibition after washout determined by the target protein turnover rate. The most developed technology in this domain are PROTACs (PROteolysis-TArgeting Chimeras), with several molecules having already reached clinical trials [7,8]. Nevertheless, the Achilles’ heel of the PROTACs is their heavy dependence on discovered small-molecule ligands on the shelf. This constraint hinders the progression toward a universal target protein degradation tool, limiting the druggable space of proteins that could be reached via such regulation. Antibodies and antibody-like proteins could be raised against different protein targets, including those without relevant small-molecule ligands. Switching from small-molecule ligands to miniproteins, such as antibody-like proteins, may alleviate the PROTACs’ drawback. The strategy of antibody-like protein employment as a binding domain for target protein degradation was explored in several articles [9,10,11,12,13], but the intracellular delivery method for possible therapeutic applications is still an issue, as it is for antibody delivery itself [14,15]. Earlier, we developed modular nanotransporter (MNT) technology, utilizing receptor-dependent endocytosis, active endosomal escape, and intracellular transport signals for targeted cargo delivery into the target cell compartment [16]. The applicability of this approach for intracellular delivery of antibody-like proteins was demonstrated using SARS-CoV-2 N-protein [17] as targets. In this study, we modified the SARS-CoV-2 N-protein targeting MNT to hijack cellular Keap1 E3-ligase for N-protein degradation. The functionality of this MNT, its cellular effects on cells expressing SARS-CoV-2 N-protein, and its ability to suppress the target were investigated.

## 2. Materials and Methods

### 2.1. Cell Lines

The adenocarcinoma human alveolar basal epithelial cells (A549) and human epidermoid carcinoma cells (A431) were maintained according to the specifications of the American Type Culture Collection (ATCC, Manassas, VA, USA).

### 2.2. SARS-CoV2 N-Protein Stable Cell Lines Generation

The plasmids from Addgene were used for lentiviral transduction. pMD2.G and psPAX2 were gifts from Didier Trono (Addgene plasmids #12259 and #12260, respectively). pHAGE N-mRuby3 (from SARS-CoV-2) IRES puro was a gift from Raphael Gaudin (Addgene plasmid #170466). Recombinant lentiviral particles were produced by cotransfection of HEK293T cells in a T25 flask with 2.3 μg pMD2.G, 4.3 μg psPAX2, and 10.2 μg pHAGE N-mRuby3 (from SARS-CoV-2) IRES puro using Calcium Phosphate Transfection Reagent (Thermo Fisher Scientific, Waltham, MA, USA). The virus suspension was collected 72 h after transfection. The lentivirus was concentrated using the Lenti-X concentrator (Takara Bio, Otsu, Japan) according to the manufacturer’s protocol. Human cancer cell lines A549 and A431 were seeded in 24-well plates (1 × 10^4^ cells/well) one day before viral infection. For lentivirus transduction, lentiviral particles were suspended in a culture medium containing 10 μg/mL polybrene (Sigma-Aldrich, Burlington, MA, USA). Stable cell lines were selected with puromycin (1 μg/mL, Acros Organics, Geel, Belgium).

### 2.3. A549 Transient Transfection with Plasmid Coding for SARS-CoV2 N-Protein

A549 cells were grown in Dulbecco’s Modified Eagle’s medium (DMEM) supplemented with 10% fetal bovine serum with 50 µg/mL gentamicin and maintained in a tissue culture incubator at 37 °C with 5% CO_2_. After passaging in a T25 flask and reaching about 70–80% confluence, cells were seeded into POCmini chambers (Cell Cultivation System: Open Cultivation, PeCon, Erbach, Germany), 50,000 cells per chamber in 1 mL of DMEM medium with 10% bovine fetal serum, and cultivated at 37 °C under 5% CO_2_. The next day, cells were transfected with pHAGE N-mRuby3 (from SARS-CoV-2) IRES puro (Addgene plasmid #170466) using Lipofectamine 3000 according to the manufacturer’s instructions.

### 2.4. Recombinant Proteins Used in the Work

**Figure 1:**
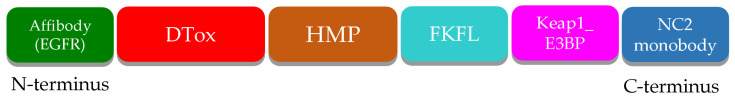
Scheme of the MNT_1_. A description of the modules is given in the text.

The main object of this study is a modular nanotransporter of the following composition (Figure 1): affibody(EGFR)-HisTag-DTox-HMP-FKFL-Keap1_E3BP-NC2, where affibody(EGFR) is an antibody mimetic that binds to the epidermal growth factor receptor (EGFR), HisTag is a peptide of six histidines, necessary for the isolation and purification of MNTs, DTox—endosomolytic module based on the translocation domain of diphtheria toxin, HMP—hemoglobin-like protein of *E. coli*, acting as a carrier module, FKFL—one of the most optimal endosomal protease cathepsin B cleavage sites [18], Keap1_E3BP is the amino acid sequence DPETGEYL, selected on the basis of the peptide PROTAC molecule proposed in article [19] and binding the ubiquitin ligase Keap1 with nanomolar affinity, and NC2—monobody NC2 to the N-protein. Further, this construct is designated as MNT_1_. In addition, a transporter that lacks Keap1_E3BP will be designated as MNT_0_. The control transporter, MNT_con_, is MNT_0_ lacking the FKFL site and NC2 monobody.

### 2.5. Protein Isolation and Purification

*E. coli* cells of the Ros(DE3)pLysS strain were transformed with the plasmid containing the resulting MNT_1_ construct. An overnight culture of these cells was seeded in 1 L of Terrific modified medium (Dia-M, Moscow, Russian Federation), with the addition of kanamycin (30 μg/mL), chloramphenicol (40 μg/mL), 2.9 g/L glucose, 7.6 g/L lactose, and 11.2 g/L glycerol. These cells were grown at 37 °C with constant stirring until an optical density of 0.8 units was reached at a wavelength of 600 nm. Autoinduction of protein expression was carried out by incubation for 48 h at a temperature of +18 °C and constant stirring. After this, the cells were separated from the medium by centrifugation for 30 min at 9000 rpm (JA-10 rotor, Beckman, Brea, CA, USA). Cell lysis was carried out for 1.5 h at 4 °C in a buffer of the following composition: 50 mM Na_2_HPO_4_, 500 mM NaCl, 5 mg/mL lysozyme, 1 cOmplete™ tablet (EDTA-free protease inhibitor cocktail (Roche, Basel, Switzerland)), 2.5 units/mL benzonase (purity > 90%, Novagen), 0.5% Triton X-100, and 10% glycerol, pH 8. The lysate was then centrifuged for 30 min at 15,000 rpm (JA-20 rotor, Beckman, USA), the supernatant was collected, and imidazole was added to it to a final concentration of 20 mM and NaCl to 500 mM, after which it was loaded onto a metal affinity chromatography column Ni HisTrap FF, 5 mL (Cytiva, Marlborough, MA, USA). The column was washed with a buffer of the following composition: 50 mM Na_2_HPO_4_, 500 mM NaCl, 25 mM imidazole, 0.5% Triton X-100, and 5% glycerol, pH 7.8. The MNT_1_ was eluted using a BioLogic LP low-pressure chromatography device (Bio-Rad, Hercules, CA, USA) in a buffer consisting of 50 mM Na_2_HPO_4_, 500 mM NaCl, and 700 mM imidazole at pH 8 (elution buffer). Fractions containing protein were dialyzed three times against a buffer of 10 mM Na_2_HPO_4_, 150 mM NaCl, pH 8.0 (PBS). The purity of the isolated MNT_1_ as well as the purity of other proteins was assessed in Image Lab 6.0.0 software (BioRad, Hercules, CA, USA) based on the results of denaturing polyacrylamide gel electrophoresis (PAGE) according to Laemmli. The purity of the resulting MNT_1_ was 86.6%.

*E. coli* strain Ros(DE3)pLysS cells were transformed with the plasmid containing the resulting MNT_0_ construct. Cells were seeded in LB Broth Miller nutrient medium (Luria-Bertani) and grown at a temperature of 37 °C with constant stirring until an optical density of 0.8 units was reached at a wavelength of 600 nm. Induction of protein expression was carried out by adding isopropyl-β-D-1-thiogalactopyranoside (IPTG) to the nutrient medium to a final concentration of 500 μM and incubating for 18 h at a temperature of +17 °C and constant stirring. After this, the cells were pelleted by centrifugation. Lysis, purification, and elution were carried out in the same way as for MNT_1_. The purity of the resulting MNT_0_ was 96%.

*E. coli* strain BL21(DE3) cells were transformed with the plasmid containing the resulting MNT_con_ construct. Production, lysis, and purification were carried out the same way as for MNT_1_. The MNT_con_ was sequentially eluted from the column with buffers containing 500 mM NaCl, 50 mM Na_2_HPO_4_, and 70 mM imidazole, pH 8; 500 mM NaCl, 50 mM Na_2_HPO_4_, and 100 mM imidazole, pH 8; 500 mM NaCl, 50 mM Na_2_HPO_4_, and 700 mM imidazole, pH 8. Fractions containing pure MNT_con_ were dialyzed 3 times against PBS buffer (10 mM Na_2_HPO_4_, 150 mM NaCl, pH 8.0). The purity of the resulting MNT_con_ was 93%.

*E. coli* cells of strain BL21(DE3), transformed with a plasmid encoding the N-protein of SARS-CoV-2, were seeded in LB medium and grown at a temperature of 37 °C and constant stirring until optical density 0.8 units at a wavelength of 600 nm. Induction of N-protein expression was carried out by adding IPTG to the nutrient medium to a final concentration of 500 μM and incubating for 3 h at a temperature of 37 °C and constant stirring. After this, the cells were pelleted by centrifugation. Cell lysis was carried out for 3 h at a temperature of +4 °C in a buffer of the following composition: 500 mM NaCl, 50 mM Na_2_HPO_4_, 10 mg/mL lysozyme, 0.5% Triton X-100, 1 mM PMSF, and 50 KIU/mL aprotinin. The inclusion body fraction was separated from the lysate by centrifugation. Inclusion bodies were washed sequentially twice with a TE50/20 solution (50 mM Tris, 20 mM EDTA, and 1 mM PMSF, pH 8.0) with the addition of NaCl and Triton X-100 to a final concentration of 0.5 M and 2%, respectively, then once with a Tris–NaCl solution (20 mM Tris, 1 M NaCl, and 1 mM PMSF, pH 8.0) with the addition of Triton X-100 to a final concentration of 2%, after which the inclusion bodies were washed with a Tris–NaCl solution (20 mM Tris, 1 M NaCl, and 1 mM PMSF, pH 8.0). The inclusion body pellet was resuspended for two hours at room temperature in a buffer containing 500 mM NaCl, 50 mM Na_2_HPO_4_, and 8 M urea, pH 7, and then centrifuged. The supernatant was applied to an affinity chromatographic column Protino^®^ Ni-TED Resin, after which the column was washed with buffers of the following composition: 500 mM NaCl, 50 mM Na_2_HPO_4_, and 1 M urea, pH 7, as well as 500 mM NaCl, 50 mM Na_2_HPO_4_, 1 M urea, and 0.06% SDS, pH 7. The N-protein was eluted from the column with a buffer containing 500 mM NaCl, 50 mM Na_2_HPO_4_, 1 M urea, and 700 mM imidazole, pH 7. The protein was dialyzed 3 times against HBS buffer (10 mM HEPES, 150 mM NaCl pH 8.0). The purity of the resulting N-protein was 91%.

After purification, all protein solutions were centrifuged and sterilized by filtration. The concentrations of the purified MNTs were measured with the Bradford method.

### 2.6. Flow Cytometry

Flow cytometry was used to study the ability of MNT_1_ to bind to EGFR receptors on the surface of A431 cells. The MNT_1_ was labeled with AF488 fluorescent dye. To do this, an 8-fold molar excess of the activated AF488-N-hydroxysuccinimide ester (Lumiprobe, Moscow, Russia) was added to the MNT_1_ in 65 mM carbonate buffer (pH 8.5), and the mixture was incubated for 1 h at room temperature with constant stirring. The MNT_1_ with the attached AF488 was purified from the unreacted dye using a PD10 gel filtration column. As a result, an average of two AF488 molecules were attached to one MNT_1_ molecule. A431 cells seeded in a 24-well plate were incubated with 500 nM MNT_1_ for a given time, then washed twice with Versene solution, then detached with a 0.25% trypsin solution in Versene, and finally resuspened in Hank’s solution. The amount of MNT_1_-AF488 bound to cells was determined using a CytoFLEX S flow cytometer (Beckman Coulter, Inc., USA) in the fluorescence channel of 500–550 nm. Fluorescence was excited with a laser with a wavelength of 488 nm. The average fluorescence value per cell was determined from 7–13 replicates at different incubation times with MNT_1_-AF488. Large cell aggregates were excluded from consideration.

To study N-protein degradation, A549 and A431 cell lines, stably transfected with plasmid coding for N-protein fused to the fluorescent protein mRuby3, were used. A431 cells or A549 cells seeded in a 24-well or a 48-well plate were incubated with MNT_1_ or MNT_0_ for a given time, then washed twice with Versene solution, then removed with a 0.25% trypsin solution in Versene, and then transferred to Hank’s solution. The average fluorescence value per cell was determined from 5–19 replicates at different incubation times with 500 nM MNT_1_ or 500 nM MNT_0_ using a CytoFLEX S flow cytometer in the fluorescence channel of 564–606 nm. Fluorescence was excited with a laser with a wavelength of 561 nm. The average fluorescence value per cell was determined from 7–13 replicates at different incubation times with MNT_1_-AF488. Large cell aggregates and cellular debris were excluded from consideration (Figure S1). The graphs were created using Origin 6.0 and GraphPad Prism 6 software. Statistical analysis was carried out using GraphPad Prism 6 software. Data were checked for normal distribution. If data failed the test for normality, the nonparametric Mann–Whitney test was used to assess the significance of the difference between two values.

### 2.7. Liposome Leakage Assay

Utilizing unilamellar phosphatidylcholine liposomes (Fluka, Seelze, Germany) loaded with the fluorescent dye calcein (Sigma-Aldrich, Burlington, MA, USA), MNT_1_′s capacity to induce liposome leakage was established. Unilamellar liposomes were loaded with fluorescent dye calcein to the fluorescence self-quenching concentration of 100 mM. Using a W-181-T sonicator (Ulta Sonic Finland Ltd., Lanti, Finland; 40 kHz, 90 W, 0 °C, 30 min), fresh lipid suspension was sonicated until clear in liposome buffer containing HEPES (20 mM), MES (20 mM), citrate (20 mM), and sodium chloride (150 mM), pH 7.4. For size standardization, the resultant liposomes were run through Durapore filters with a pore size of 0.22 µm (Millipore, Burlington, MA, USA) for size standardization ten times. The liposomes were kept in an argon environment at 4 °C. Before the experiment, liposomes were purified on PD-10. They were then treated for 30 min in triplicate at room temperature with 100 nM MNT_1_ in liposome buffer at various pH levels. After that, samples were diluted tenfold in liposome buffer, pH 7.5, and the fluorescence of free calcein (leaked from liposomes) was measured at 520 nm (excitation at 490 nm). Samples with 0.5% Triton X-100 were utilized as a positive control (100% calcein leakage). Parallel samples with MNT1 deleted were used to measure background calcein leakage.

### 2.8. Thermophoresis

The interaction affinity between MNT_1_ or cleaved MNT_1_ and N-protein was measured with a Monolith NT.115 instrument (NanoTemper Technologies, München, Germany) in phosphate buffer (25 mM NaH_2_PO_4_, 150 mM NaCl, pH 8.0). The N-protein was labeled with the AF488 fluorescent dye. To do this, a 4-fold molar excess of the activated AF488-N-hydroxysuccinimide ester was added to the N-protein in 65 mM carbonate buffer (pH 8.5), and the mixture was incubated for 1 h at room temperature with constant stirring. The N-protein with attached AF488 was purified from the free unreacted dye via gel filtration on PD10 chromatographic column. As a result, an average of 2.2 AF488 molecules per one N-protein molecule modification was obtained. At a fixed concentration of N-protein-AF488 (5 nM), thermophoresis curves were obtained. Four such curves were obtained for each experiment, and the whole experiment was repeated three or four times. For each curve, the dissociation constant of the N-protein complex with MNT_1_ or cleaved MNT_1_ was determined with Monolith NT.115 Instruments software, then the constant was averaged over all 14–17 curves, and the relative measurement error was determined. Cleaved MNT_1_ was prepared by incubating 4 μM MNT_1_ with 4 μg/mL activated native human cathepsin B (ab90387, Abcam, Waltham, MA, USA) for 20 h at 37 °C. Activation of cathepsin B was carried out as described by Kern et al. [20]. The graphs were created in Origin 6.0 software.

### 2.9. Western Blot

N-protein degradation was studied in A549 and A431 cells stably transfected with plasmid coding for N-protein fused to the fluorescent protein mRuby3 using western blots with antibodies to the N-protein. Cells were harvested via trypsin treatment and centrifugation (200× *g*, 5 min). Then cells were suspended in a buffer (pH 8.0) containing 25 mM NaH_2_PO_4_, 150 mM NaCl, cOmplete, (EDTA free protease inhibitor cocktail, 11873580001, www.sigmaaldrich.com, accessed on 1 September 2023), and 10 mM EDTA. The number of cells was quantified using a CytoFLEX S flow cytometer. The cells were lysed by four freezing–thawing cycles. Freezing was performed in liquid nitrogen. Then, the cell lysates were centrifuged (10,000× *g*, 5 min). The supernatants of the cell lysates underwent denaturing electrophoresis, followed by a western blot stained with anti-N-protein mAb (SARS-CoV-2 Nucleocapsid Polyclonal Antibody, PA5-116894, Invitrogen, Carlsbad, CA, USA) and secondary antibodies goat-antirabbit + Peroxidase (G21234, Thermo Fisher Scientific). Sample electrophoresis was performed using standard 10% SDS-PAGE. Given the frequent inhomogeneous coloration of samples obtained using western blotting, all samples were loaded on the gel in either duplicate or triplicate. This enables us to exclude random outliers of individual bands from the analysis and to obtain more reliable averaged data. Samples were transferred from the gel to a supported nitrocellulose membrane (0.22 μm) using the Trans-Blot Turbo Transfer System (Bio-RAD) (Figures S2 and S3). For each sample in the Image Lab 6.0.0 software, the average signal intensity was measured in the area where the band corresponding to the N-protein was observed and where it was not observed (Figure S2), and they were subtracted from each other. Next, the intensity of each band was normalized to the cell concentration in the corresponding sample, which was determined using flow cytometry. The resulting normalized intensities were in turn normalized to the intensity of the control, which was the sample to which the modular nanotransporter was not added. When inhibitors were used, only the desired inhibitor was added to the corresponding control. Four series of experiments were performed, in each of which the incubation time with MNT varied. For each series, one or two western blots were obtained, on each of which a separate time point sample was applied in 2–3 replicates (Figures S2 and S3). A single time point resulted in 6–19 replicates. Unstained bands were excluded (they were observed mainly along the edges of the gel). In A431 cells, the effect of the proteasomal degradation inhibitor MG-132 (S2619, Selleckchem.com, Houston, TX, USA) and the autophagy inhibitor Bafilomycin A1 (54645S, Cell Signaling Technology, Danvers, MA, USA) on N-protein degradation was also studied (Figure S4). To do this, MNT_1_ was incubated for 24 h with 5 μM MG-132 or 100 nM Bafilomycin A1. Next, the relative amount of N-protein in each cell sample was determined using western blotting in *n* = 8–15 replicates. The graphs were created in the Origin 6.0 and the GraphPad Prism 6 programs. Statistical analysis was carried out using the GraphPad Prism 6 program. Data were checked for normal distribution. If the test for normality did not pass, then the nonparametric Mann–Whitney test was used to assess the significance of the difference between two values.

### 2.10. Confocal Microscopy

Images of A549 cells transiently transfected with plasmid coding for N-protein fused to the fluorescent protein mRuby3 were obtained on a confocal microscope STELLARIS 5 (Leica, Wetzlar, Germany) using a 63× objective with NA = 1.3. Excitation of mRuby3 fluorescence was carried out using a laser with a wavelength of 561 nm, and fluorescence registration was carried out in the range of 580–640 nm. Nuclei were stained with the fluorescent dye Hoechst 33342. This dye was excited with a laser with a wavelength of 405 nm, and its fluorescence was recorded in the range of 415–420 nm. For the mRuby3 fluorescence channel, the cell cytoplasm region was selected in the ImageJ Fiji 1.54f software, and then the cell-average standard deviation of fluorescence intensity was calculated. Next, such standard deviations were averaged over all cells, and the standard deviation of noise signal, which was calculated in areas where there are no cells, was subtracted from the resulting value.

## 3. Results

**Figure 2:**
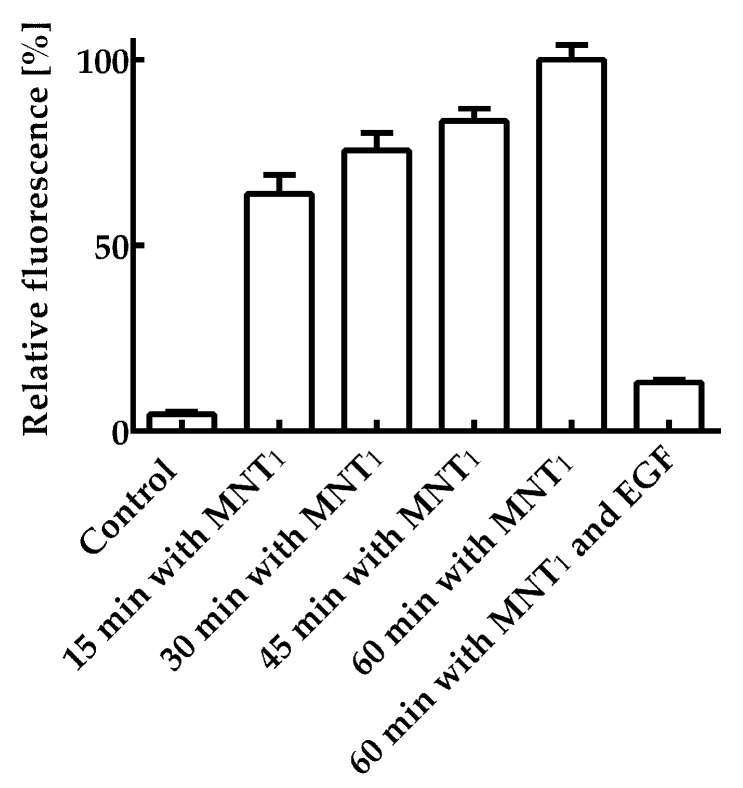
Interaction of AF488 fluorescently labeled MNT_1_ with EGR-positive A431 cells. A control group without MNT_1_-AF488 addition, groups in which 200 nM MNT_1_-AF488 were incubated with cells for 15, 30, 45, and 60 min, and a group in which 200 nM MNT_1_-AF488 in the presence of 2 μM EGF was incubated with cells are shown. The fluorescence of the cells incubated with MNT_1_-AF488 for 60 min was taken as 100%. Mean values are given ± 95% confidence intervals (*n* = 7–13).

First of all, it was necessary to study the retention of the functional activities of each module within the MNT_1_. The ability of the AF488 labeled MNT_1_ labeled to bind to its receptors was studied on the A431 cells. Figure 2 shows the average fluorescence values per cell for various incubation times of 200 nM MNT_1_-AF488 with A431 cells. The average cell fluorescence after 15 min of incubation was significantly (*p* < 0.05, ANOVA multiple comparisons, nonparametric test) higher than that of the control, to which MNT_1_-AF488 was not added. To verify that the internalization of MNT_1_, containing an affibody to the epidermal growth factor receptor (EGFR), is EGFR-specific, a control experiment was performed in which A431 cells were first incubated for 1 h with 1 μM epidermal growth factor, EGF, and then these cells were incubated with 200 nM MNT_1_-AF488, to which EGF was added at a concentration of 2 μM. In this case, the average fluorescence significantly (*p* < 0.05, ANOVA multiple comparisons, nonparametric test) decreased (Figure 2). Thus, one can conclude that the internalization of MNT_1_ into A431 cells occurs mainly in an EGFR-dependent manner; i.e., the affibody in MNT_1_ retains its functional activity.

**Figure 3:**
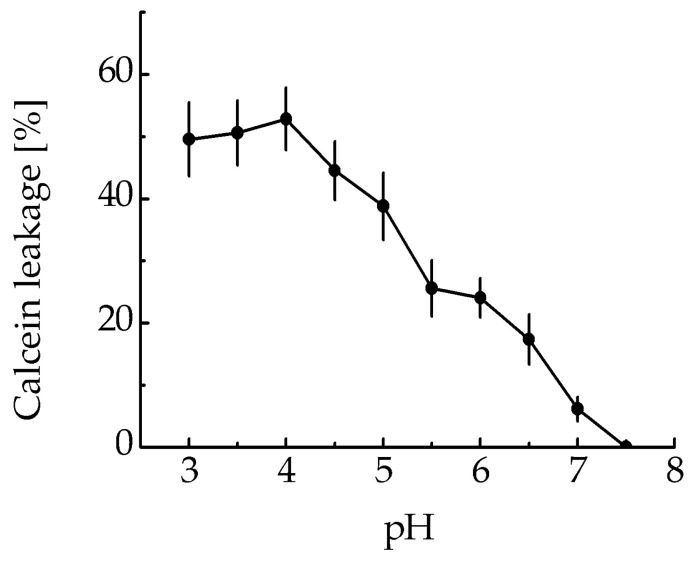
MNT_1_-induced leakage from egg yolk phosphatidylcholine liposomes loaded with the fluorescent dye calcein at the fluorescence self-quenching concentration. The appearance of fluorescence indicates liposome leakage. Error bars are SEM (*n* = 3).

The membranolytic activity of the MNT_1_ was assessed by testing MNT1’s ability to cause leakage of calcein-loaded liposomes at various pHs. The MNT1-mediated liposome leakage was detected in acidic pHs, including the range characteristic for endosomal pHs from 5–6 (Figure 3), while almost no leakage was observed at neutral pHs (Figure 3). Thus, the endosomolytic module within MNT_1_ retains its functional activity.

**Figure 4:**
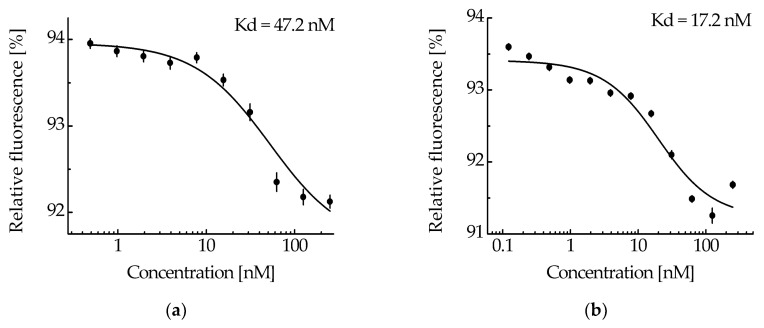
The interaction of MNT_1_ (**a**) and cleaved MNT_1_ (**b**) with N-protein-AF488 assessed by thermophoresis. Dependences of relative fluorescence intensities (fluorescence intensity before the start of thermophoresis is taken as 100%) at 20 s after the start of thermophoresis on the concentration of the MNT_1_ (**a**) or cleaved MNT_1_ (**b**) at a constant concentration of the N-protein-AF488 (5 nM). Standard errors (SE) of relative fluorescence intensities are shown (*n* = 14–17). The equilibrium dissociation constant of the MNT_1_ or cleaved MNT_1_ complex with the N-protein, Kd, is indicated.

The ability of the NC2 monobody in the MNT_1_ molecule to bind to the N-protein was tested by thermophoresis. Based on the obtained dependence of relative fluorescence on MNT_1_ concentrations at a constant concentration of N-protein (Figure 4a), the equilibrium dissociation constant of the complexes of MNT_1_ with N-protein was 47.2 ± 2.5 nM (±SE). Thus, the NC2 monobody retains its functional activity as part of MNT_1_.

**Figure 5:**
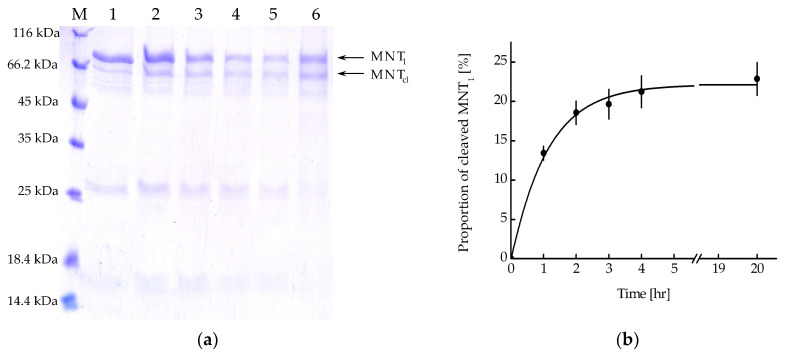
(**a**) Availability of the cathepsin B cleavage site in MNT_1_. Cleavage of MNT_1_ (4 μM) with cathepsin B (4 μg/mL); polyacrylamide gel electrophoresis. Samples 1—MNT_1_ without cathepsin B, 2—1 h of incubation of MNT_1_ with cathepsin B, 3—2 h of incubation of MNT_1_ with cathepsin B, 4—3 h of incubation of MNT_1_ with cathepsin B, 5—4 h of incubation of MNT_1_ with cathepsin B, 6—20 h of incubation of MNT_1_ with cathepsin B. M—protein standards. MNT_1_—original MNT_1_, MNT_cl_—cleaved MNT_1_. (**b**) Kinetics of cleavage of MNT_1_ (4 µM) by the endosomal protease cathepsin B (4 µg/mL) at pH 5.5. Shown means ± SE (*n* = 4–5).

MNT_1_ includes the FKFL site, which allows cathepsin B to cleave the NC2 monobody and the sequence for binding the Keap1 E3 ubiquitin ligase. Figure 5a shows the kinetics of this cleavage upon the addition of activated cathepsin B. From the kinetic curve of cleavage obtained from analysis of the intensity of the band corresponding to cleaved MNT_1_, it can be seen that after 4 h of incubation of MNT_1_ with cathepsin B, the proportion of cleaved MNT_1_ reaches a plateau (Figure 5b). Interpolating this curve (Figure 5b) with the dependence y = a × (1 − exp(−k × t)), where a is the maximum proportion of cleaved MNT_1_, and k is the cleavage rate constant, k was 0.89 ± 0.07 h^−1^. Thus, in MNT_1_, the FKFL site is accessible for cleavage via the endosomal protease cathepsin B.

MNT_1_ (4 μM), which was incubated for 20 h with cathepsin B (4 μg/mL), was considered the most cleaved. At a fixed concentration of N-protein-AF488 (5 nM), thermophoresis was used to obtain the dependence of the relative fluorescence on the concentration of cleaved MNT_1_ (Figure 4b). The dissociation constant of the NC2 monobody complex with N-protein-AF488 determined from this dependence was 17.2 ± 4.4 nM, compared to 47.2 ± 2.5 nM for the full-sized MNT_1_ (Figure 4). Thus, if the NC2 monobody with Keap1_E3PB is cleaved from MNT_1_ in endosomes, then its affinity for N-protein increases.

**Figure 6:**
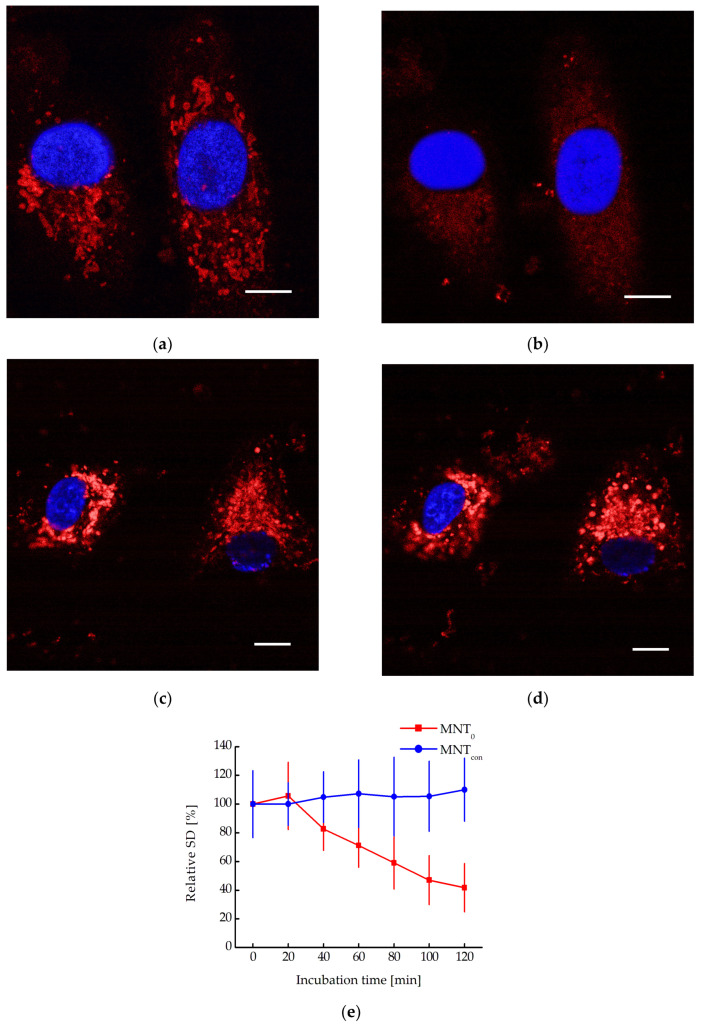
Confocal microscopy images of A549 cells transiently transfected with plasmid encoding N-protein fused to the red fluorescent protein mRuby3 and incubated with 500 nM MNT_0_ for 0 min (**a**) and 80 min (**b**) or with 500 nM MNT_con_ for 0 min (**c**) and 80 min (**d**). Blue shows the staining of nuclei with Hoechst 33342 dye. Bar is 10 μm. (**e**) Dependence of the relative standard deviation of fluorescence intensity in the cytoplasm (the standard deviation of fluorescence intensity at the initial time point is taken as 100%), SD, on the time of incubation of A431 cells with 500 nM MNT_0_ or with 500 nM MNT_con_. The background SD was subtracted. Shown means ± SD.

We have previously shown that MNT_0_ can interact with N-protein in A431 cells transiently transfected with plasmid encoding N-protein fused to the fluorescent protein mRuby3 [17]. It is known that in cells, N-protein causes liquid–liquid phase separation with the formation of so-called biocondensates [21,22]. The destruction of these biocondensates significantly affects both the stability of the N-protein and the viral capsid assembly, ultimately leading to the suppression of virus replication [21,22]. Biocondensate-like structures are observed in A431 cells transiently transfected with a plasmid coding for N-protein fusion to mRuby3 (Figure 6). Moreover, incubation of these cells with MNT_0_ leads to their destruction and the appearance of more uniform fluorescence (Figure 6a,b). In contrast, incubation with the control MNT, MNT_con_, does not lead to the destruction of these structures (Figure 6c,d). To quantitatively describe this process, the standard deviation of fluorescence intensity in the cytoplasm can be used [23]. Indeed, when cells are incubated with MNT_0_, this standard deviation decreases, while when cells are incubated with MNT_con_, it does not change (Figure 6e). Moreover, starting from 100 min of incubation, the data for MNT_0_ and MNT_con_ differ significantly (*p* < 0.05, Mann–Whitney test). Thus, the interaction of the studied MNTs with the N-protein in the cell leads to the destruction of the N-protein biocondensates.

**Figure 7:**
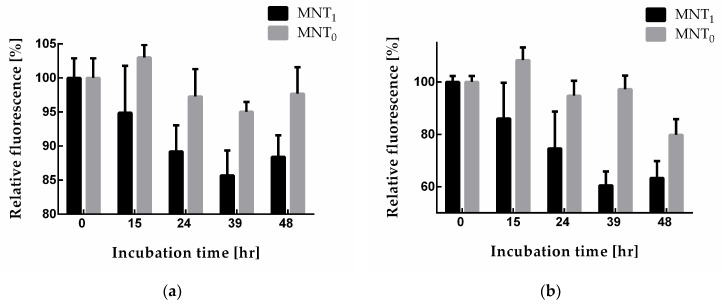
Relative fluorescence of A549 (**a**) and A431 (**b**) cells (the fluorescence of cells incubated without MNT was taken as 100%) when they were incubated for different times with either 500 nM MNT_1_ or 500 nM MNT_0_. Shown means ± SD.

The N-protein degradation in A549 and A431 cells stably transfected with plasmid encoding N-protein fused with the red fluorescent protein mRuby3 can be observed with a decrease in the fluorescence of this protein. To do this, cells were incubated with MNT_1_ and MNT_0_ for different time intervals and washed, and the average mRuby3 fluorescence per cell was measured using flow cytometry. Figure 7 shows the obtained dependences of cell fluorescence on the time of cell incubation with these MNTs. Starting from 15 h of incubation, the cells incubated with MNT_1_ exhibit significantly (*p* < 0.05, Mann–Whitney test) diminished fluorescence compared to that of the cells incubated with MNT_0_ (Figure 7). Thus, the presence of a sequence that recruits the Keap1 E3 ligase in MNT_1_ leads to a noticeable decrease in the fluorescence of the mRuby3 fused to the N-protein, which may indicate degradation of the N-protein.

**Figure 8:**
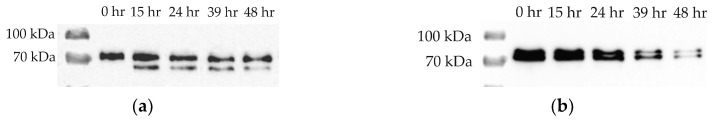

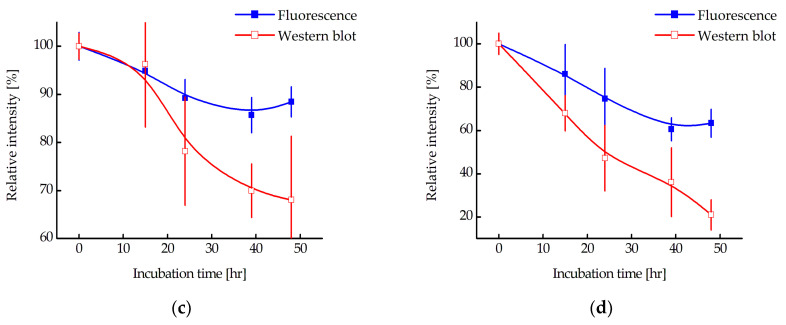
Western blot with N-protein antibodies for lysates of A549 (**a**) and A431 (**b**) cells stably transfected with plasmid encoding N-protein fused with mRuby3. Cell incubation times with 500 nM MNT_1_ are indicated. Relative intensity of fluorescence or western blot band for N-protein for A549 (**c**) and A431 (**d**) cells (the intensity of fluorescence or western blot band for cells to which MNT was not added was taken as 100%) when they were incubated for different times with 500 nM MNT_1_. Western blot data were normalized to the cell concentration in the corresponding sample, which was determined using flow cytometry. Shown means ± SD.

Since a decrease in fluorescence does not directly indicate degradation of N-protein, the relative amount of N-protein in the cell during incubation with MNT_1_ was also measured using western blotting (Figure 8a,b). The intensities of the corresponding bands were normalized to the cell concentration and to the intensity of the band corresponding to cells to which MNT_1_ was not added. Figure 8c,d shows a comparison of the cell fluorescence data to the western blot data for A549 and A431 cells, respectively. It can be seen that the relative amount of N-protein indeed decreases greatly, especially for the A431 cells (Figure 8d), and noticeably faster than the fluorescence of the cells. Moreover, at 48 h incubation for A549 cells and starting from 15 h of incubation for A431 cells, the western blot data significantly differed from the cell fluorescence data (*p* < 0.01, Mann–Whitney test). Thus, MNT_1_ causes degradation of the N-protein in A549 and A431 cells stably transfected with plasmid encoding N-protein fused to mRuby3. Cell fluorescence also decreases, but it does not reflect the entire proportion of N-protein that has undergone degradation.

**Figure 9:**
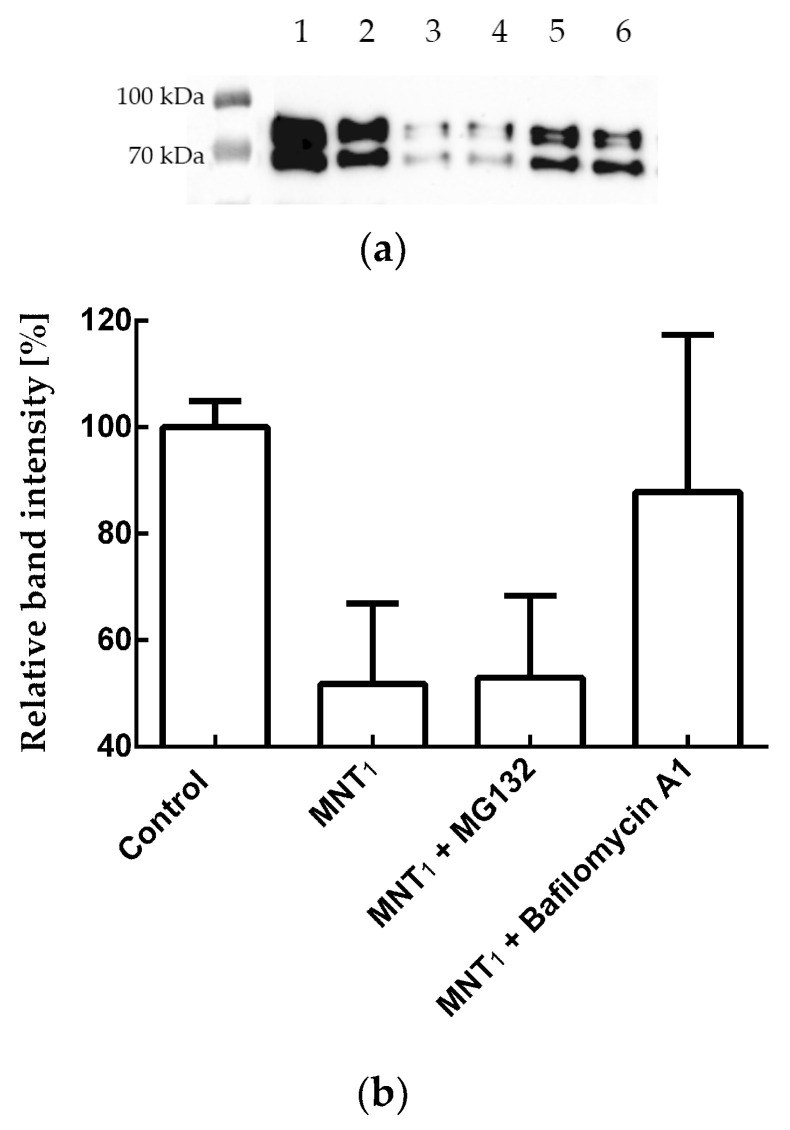
(**a**) Western blot with N-protein antibodies for lysates of A431 cells stably transfected with plasmid coding for N-protein fused to the fluorescent protein mRuby3. Samples shown: 1—A431 cells to which MNT_1_ and inhibitors were not added, 2—A431 cells that were incubated with 500 nM MNT_1_ for 24 h, 3—A431 cells that were incubated with 5 μM MG132 for 24 h, 4—A431 cells that were incubated with 500 nM MNT_1_ and 5 μM MG132 for 24 h, 5—A431 cells that were incubated with 100 nM Bafilomycin A1 for 24 h, and 6—A431 cells that were incubated with 500 nM MNT_1_ and 100 nM Bafilomycin A1 for 24 h. (**b**) The influence of MG132 and Bafilomycin A1 on MNT_1_-induced degradation of N-protein. Relative intensity of western blot band for the N-protein for A431 cells (the intensity of western blot band for cells to which MNT was not added, but containing the appropriate inhibitor, was taken as 100%) when they were incubated for 24 h with MNT_1_ (500 nM) and MNT_1_ (500 nM) with MG132 (5 μM) or Bafilomycin A1 (100 nM). Shown means ± SD. Western blot data were normalized to the cell concentration in the corresponding sample, which was determined using flow cytometry.

To determine the pathway of the N-mRuby3 fusion protein degradation, we used the proteasomal degradation inhibitor MG132 and the autophagy inhibitor Bafilomycin A1. Figure 9 shows that for A431 cells, the addition of MG132 together with MNT_1_ does not lead to significant inhibition of N-protein degradation (*p* > 0.05, Mann–Whitney test). In contrast, the addition of Bafilomycin A1 together with MNT_1_ causes significant inhibition (*p* < 0.05, Mann–Whitney test) of degradation of the N-mRuby3 fusion protein (Figure 9). Thus, the degradation of the N-mRuby3 protein occurs mainly via the autophagy pathway.

## 4. Discussion

The field of induced degradation of viral target proteins is just beginning to develop; however, a number of studies demonstrating the fundamental feasibility of this approach have already been published. Thus, in the work of de Wispelaere et al. [24], a proteolysis targeting chimera (PROTAC) based on a hepatitis C virus NS3/4A protease inhibitor led to target degradation and inhibition of viral infection in cells. At the same time, the compound proposed by the authors was also capable of degrading NS3/4A protease mutants, which were resistant to the action of the original inhibitor on the basis of which the PROTAC was obtained [24]. A similar ability to overcome drug resistance to the parent inhibitor has been described for PROTACs targeting cancer cells [25,26]. In the case of the influenza virus, PROTAC molecules were created that used both neuraminidase [27] and hemagglutinin [28] as targets. A natural antiviral PROTAC that recruits the E3 ligase TRIM25 to influenza virus polymerase has also been described [6]. At the same time, PROTAC, created on the basis of the oseltamivir inhibitor, had an antiviral effect against both the wild-type and oseltamivir-resistant strains [27]. The idea of using a PROTAC approach to create an anti-coronavirus drug was proposed at the beginning of the pandemic [29]. However, besides our work, only one work with experimental data on the PROTAC approach for the purpose of suppressing SARS-CoV-2 was published at the end of November 2023 as a preprint [30]. In this work, the authors created a PROTAC based on a SARS-CoV-2 protease inhibitor and demonstrated both target protein degradation and the resulting antiviral activity on cells [30]. In addition to academic research, pharmaceutical companies are also working on developing means to induce the degradation of coronavirus proteins [31].

An important drawback of PROTAC approaches is their dependence on engineered small-molecule ligands—usually inhibitors—for their targets. In turn, low-molecular-weight ligands are not a universal tool of influence and are mainly selected for enzymes, ion channels, and receptors. If the target protein does not have a suitable binding pocket, the ability to select a low-molecular-weight ligand will be greatly limited. All this hinders progress towards the creation of a universal direct approach to the degradation of target proteins based on classical PROTAC technology and limits the list of proteins that can be subject to such regulation. At the same time, antibodies and antibody-like molecules can be obtained against various antigens, including those that are inaccessible to inhibitors. At the same time, antibodies are a much more flexible tool and can be selected for various post-translational modifications, proteins with different splicing variants, or different isoforms.

The transition from small-molecule ligands to antibody-like proteins as the antigen recognition part can overcome the disadvantage of PROTAC described above. Works using antibody-like molecules to attract E3 ligase to the target protein are described in the literature [9,10,11,12,13], but they mainly use transfection or electroporation as a method of delivering the construct into a cell. This is convenient for solving fundamental problems but has significant difficulties when considering possible medical applications of this type of technology. In general, the problem of intracellular delivery of PROTACs based on antibody-like molecules has the same possible solutions as the problem of intracellular delivery of antibodies themselves [14].

To deliver antibody-like molecules, we used the technology of modular nanotransporters (MNTs) that we had previously developed [16]. These transporters can bind to an internalizable receptor on the surface of target cells using a ligand module. Then they are internalized and enter endosomes, from which they exit due to the endosomolytic module. Finally, they are transported to a selected compartment inside the cell and/or to a selected target protein using an intracellular targeting and/or effector module. All modules are combined together with a carrier module, to which the delivered cargo can also be attached. We have previously used modular nanotransporter technology to deliver antibody-like molecules targeting c-Myc [32] to target cells.

In this work, the ligand module of an MNT was an antibody-like molecule, affibody, to the human epidermal growth factor receptor (EGFR) [33,34]. The *E. coli* hemoglobin-like protein acted as a carrier module, and the translocation domain of diphtheria toxin acted as an endosomolytic module. The target protein was the nucleocapsid protein (N-protein) of the SARS-CoV-2 virus. The binding of the developed MNTs to this protein was carried out by another antibody mimetic, monobody NC2 [35]. According to the literature data, this monobody is characterized by the highest affinity for the N-protein, when compared to similar monobodies (equilibrium dissociation constant of the NC2 complex with the N-protein equals 6.7 nM) [35]. The proposed MNTs can not only bind to the N-protein but also provide its degradation due to the presence of an amino acid sequence recruiting the Keap1 E3 ligase. The key function of Keap1 is normoxia maintenance, regulating the Nrf2 level, and activating cellular antioxidant defense systems [36,37]. By fusing the Keap1-binding sequence to the N-protein recognizing monobody, we alter the Keap1 E3 ligase substrate specificity; thus, the binding of these MNTs to the N-protein should lead to ubiquitination of the N-protein and its further degradation. To increase the efficiency of the N-protein targeting part of MNT, consisting of the NC2 monobody fused with a site recruiting E3 ligase, the possibility of its endosomal cleavage via endosomal protease cathepsin B was introduced. For this, we previously selected the most optimal cleavage site for this protease [18]. The kinetics of cleavage of short peptide sequences were studied, which potentially, based on the literature data, can be cleaved by cathepsin B [18]. It was shown that the FKFL and FRRG sequences are the most quickly cleaved at endosomal pH (pH 6.0), with FKFL cleaved three times faster than FRRG [18]. Moreover, the FKFL sequence is cleaved eightfold less efficiently at pH 7.5 (outside the cell) than at pH 6.0 (in endosomes) [18], so it is assumed that the cleavage of the resulting MNT will occur mainly in endosomes. Presumably, this cleavage should improve the release of the N-protein targeting part of MNT from endosomes and increase its affinity for the N-protein. To simplify reading the MNT where its N-protein targeting part consists of NC2 monobody fused with E3 ligase recruiting site was designated MNT_1_, and a similar MNT, but lacking a site for E3 ligase was designated MNT_0_.

First of all, it was necessary to establish whether all modules within MNT_1_ retain their functional properties. The ability of the ligand module, the anti-EGFR affibody, to bind to its receptor was tested using flow cytometry for MNT_1_ labeled with a fluorescent dye. By blocking EGFR receptors with excess epidermal growth factor, it was shown that the affibody, as part of MNT_1_, retains its functional activity. The increase in the average fluorescence of A431 cells upon the addition of MNT_1_ labeled with a fluorescent dye is mainly associated with the receptor-specific binding of MNT_1_ to EGFR and the further internalization of their complex.

The preservation of the ability of the endosomolytic module to cause defects in the lipid membrane was tested at different pH values by the release of the fluorescent dye calcein from liposomes loaded with it to the fluorescence self-quenching concentration. As we showed earlier, two peaks should be observed on the curve of this release, one corresponding to the membranolytic activity of HMP (pH less than 5), and another to the membranolytic activity of the endosomolytic module DTox (pH 5.5–6) [16]. For MNT_1_, a second peak at pH 5.5–6 is also observed (Figure 3), which means that endosomolytic module DTox retains its functional activity within this transporter.

The functional activity of monobody NC2 within MNT_1_ was tested using thermophoresis. The equilibrium dissociation constant of the MNT_1_ complex with N-protein was 47.2 ± 2.5 nM. Thus, the NC2 monobody within MNT_1_ retains its ability to bind to the N-protein of the SARS-CoV-2 virus.

To test whether the FKFL site is accessible for binding and subsequent cleavage by the endosomal protease cathepsin B, the kinetics of MNT_1_ cleavage by the activated protease cathepsin B was studied in solution at pH 5.5 (Figure 5). It turned out that cleavage kinetics is quite fast, with a cleavage rate constant equal to 0.89 ± 0.07 h^−1^. Thus, the FKFL site within MNT_1_ is accessible to the cathepsin B protease, and in endosomes, the NC2 monobody with the E3 ligase Keap1 recruiting site can be cleaved from MNT_1_.

The NC2 monobody within MNT_1_ and the free monobody possess different affinities for the N-protein. Indeed, according to the literature, the equilibrium dissociation constant of the NC2 monobody complex with the N-protein of the SARS-CoV-2 virus is 6.7 nM [35], whereas within the MNT_1,_ its constant is 47.2 ± 2.5 nM. If MNT_1_ is subjected to cleavage by cathepsin B, the resulting dissociation constant hits 17.2 ± 4.4 nM. A similar post-cleavage increase in affinity was observed for MNT_0_, lacking a site for E3 ligase [17]. The equilibrium dissociation constants of the MNT_0_ or cleaved MNT_0_ complex with the SARS-CoV-2 N-protein were 116 ± 20 and 10 ± 3 nM, respectively [17]. Based on this, we can suppose that when the monobody NC2 is released from MNT in endosomes its affinity to the N-protein increases noticeably.

MNTs can interact with N-protein not only in vitro but also in cells. For example, the interaction between MNT_0_ and the N-protein was demonstrated in A431 cells transiently transfected with plasmid encoding N-protein fused to the red fluorescent protein mRuby3 using a cellular thermal shift assay [17]. For certain types of proteins, in particular the nucleocapsid protein of SARS-CoV-2, so-called liquid-liquid phase separation is observed in cells [21,22]. In this case, drop-shaped structures called biocondensates are formed, in which the concentration of the proteins responsible for their formation is significantly higher than in the rest of the solution [38]. For viruses, these biocondensates play an important role in acting as viral factories, preserving viral proteins from degradation and, on the other hand, serving as scaffolds for the construction of the viral capsid [21,22]. Previously, it was shown that simply destroying such structures by some molecule interacting with a given protein can lead to blocking the production of the virus in the cell [21]. In the present work, we observed biocondensate-like structures in A431 cells transiently transfected with plasmid encoding N-protein fused to mRuby3 (Figure 6). These structures behave differently depending on whether these cells are incubated with MNT_0_ or control MNT_con_ (Figure 6). Indeed, upon MNT_con_ addition, the biocondensates do not change; on the contrary, upon MNT_0_ addition, they start to disappear. To quantitatively describe this process, we used the standard deviation of fluorescence intensity in the cytoplasm. This standard deviation did not change in cells incubated with MNT_con_ but decreased markedly in cells incubated with MNT_0_ (Figure 6e). Thus, even the modular transporters lacking the site for the E3 ligase, MNT_0_, can cause the destruction of biocondensates formed by the N-protein, thereby preventing the formation of the viral capsid and probably increasing the rate of N-protein degradation.

The degradation of the N-protein can be conveniently monitored with the fluorescence of the mRuby3 protein fused to it. For this purpose, we obtained A549 and A431 cells stably transfected with plasmid encoding N-protein. Using flow cytometry, the fluorescence of A549 and A431 cells following incubation with MNT_1_ and MNT_0_ at various times was studied (Figure 7). Both of these proteins are able to interact with the N-protein. As mentioned above, this interaction itself can cause the destruction of biocondensates formed by the N-protein, which in turn can lead to an increase in the degradation of this protein. Indeed, for all MNTs, a decrease in cell fluorescence is observed. However, after 15 h of incubation, this decrease for MNT_1_ is significantly greater than for MNT_0_ (Figure 7). This difference is most prominent in A431 cells (Figure 7b). Thus, the MNT that is able to bind to the N-protein and contains a site for E3 ligase induces more prominent degradation of the target N-mRuby3protein compared to the MNT lacking this site.

To prove that we observed actual N-protein degradation and not just a decrease in mRuby3 fluorescence, a western blot with N-protein antibodies was used (Figure 8a,b). We showed that N-protein degradation was indeed observed, and at late incubation times, the proportion of degraded protein according to the western blot data is significantly greater than according to flow cytometry (Figure 8). In other words, fluorescence does not reflect the entire proportion of degraded N-protein. To understand why this might be related, we used inhibitors of proteasomal and autophagy degradation. For this purpose, a common inhibitor of proteasomal degradation, MG132 [39], and an autophagy inhibitor, Bafilomycin A1 [40], were selected. It turned out that Bafilomycin A1, in contrast to MG132, leads to inhibition of N-mRuby3 protein degradation (Figure 9). Based on these data, it can be assumed that N-protein degradation occurs predominantly through the autophagy pathway rather than the proteasomal pathway. It is known that ubiquitinylation of proteins can lead not only to their proteasomal degradation but also to autophagy [41,42,43]. Moreover, for fluorescent proteins, it is known that, as a result of autophagy, they are not completely degraded [44,45]. If such a fragment remains after mRuby3 degradation and fluoresces, then this could explain the difference we observed between the cell fluorescence and western blot data (Figure 8c,d).

**Figure 10:**
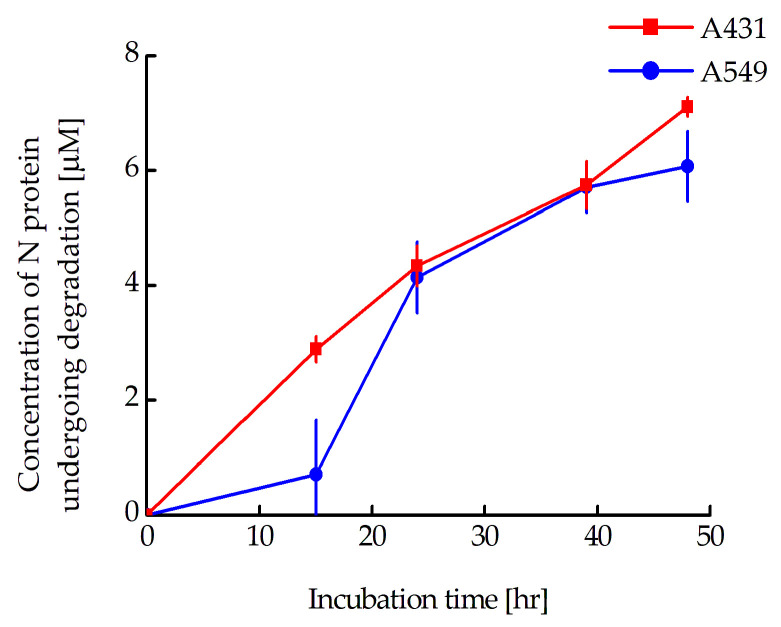
Relationships between the concentration of degraded N-protein and incubation time in A549 and A431 cells stably transfected with plasmid coding for mRuby3-fused N-protein. The concentration of degraded N-protein was calculated from the fraction of N-protein remaining in the cells obtained from western blot data. Shown means ± SE (*n* = 6–19).

According to the western blot data, there is a significant difference in the proportion of N-protein that has undergone degradation for the A549 and A431 cells. In a paper being prepared for publication, we assessed the concentrations of the N-mRuby3 protein in A431 and A549 cells stably transfected with plasmid encoding this protein. They were 9.0 ± 1.8 and 19.0 ± 1.3 µM for A431 and A549 cells, respectively. In other words, if we plot the change in the concentration of N-protein undergoing degradation at the time of incubation with MNT_1_ for A431 and A549 cells, they will coincide well with each other (Figure 10). Thus, the rate of N-protein degradation for these two cell lines is the same and is about 130 nM per hour. It should be noted that, as we previously estimated, the concentration of Keap1 does not depend on the cell type and is approximately 270 nM [46]. Moreover, the concentration of Keap1, which is capable of interacting with MNT_1_, will be significantly lower. Considering that the rate of N-protein degradation for A431 and A549 cells is the same, we can assume that the extent of its degradation is limited not by the concentration of MNT_1_ but by the concentration of Keap1, which is capable of interacting with this MNT_1_.

Our proposed approach has so far been tested only on a model of viral infection and not on the viral infection itself. In the future, we intend to study the suppression of the SARS-CoV-2 virus using the modular nanotransporters we have developed. In addition, it is known that the N-proteins of different coronaviruses possess a degree of homology of more than 90% [47]. As we have shown previously, three of the seven monobodies capable of interacting with the SARS-CoV N-protein are also capable of interacting with the SARS-CoV-2 N-protein [48]. Thus, this developed modular nanotransporter may also be effective in the treatment of SARS-CoV and MERS coronaviruses. Moreover, our proposed approach cans be extended to treat other viral diseases. It can be realized, for example, by obtaining an antibody-like molecule capable of interacting with a viral protein that is a key player for virus assembly/replication and subsequent inclusion of this antibody-like molecule into the modular nanotransporter we have developed.

## 5. Conclusions

The area of targeted degradation of viral proteins is only at the very beginning of development. Most studies on this topic use small-molecule inhibitors that can only be selected for a limited range of target proteins. In contrast, antibodies or antibody-like molecules can be produced for any selected protein. However, in the case of intracellular proteins, the problem of delivering antibody-like molecules to these proteins arises. To do this, we used the technology of modular nanotransporters that we developed, which allows us to deliver the selected molecule to the desired compartment of target cells. The modular nanotransporter obtained in the work contained an antibody mimetic molecule, affibody to EGFR, an endosomolytic module, a sequence recruiting Keap1 E3-ligase, and another antibody mimetic molecule, NC2 monobody, to the SARS-CoV-2 N-protein. All of these modules retain their functional activity as part of the proposed nanotransporter. This nanotransporter contains a cleavage site for the endosomal protease cathepsin B, which can lead to cleavage of the monobody in endosomes and a noticeable increase in its affinity for the selected viral protein, as was shown in the work for the NC2 monobody against the N-protein of the SARS-CoV-2 virus. The developed modular nanotransporters were able to induce the degradation of a selected viral protein in cells with stable expression of this protein. In the future, such nanotransporters can be used not only for the treatment of SARS-CoV-2 and other related viral diseases. By choosing the appropriate antibody mimetic to the viral protein, as well as the desired internalizible ligand for binding to the surface of target cells, their use can be extended to a wide range of viral diseases.

## Data Availability

Data are contained within this article and its Supplementary Materials.

## Supplementary Materials

The following are available online at https://www.mdpi.com/article/10.3390/pharmaceutics16010004/s1, Figure S1: Analysis of A431 (a) and A549 (b) cells stably transfected with plasmid coding for N-protein fused to the fluorescent protein mRuby3 using SSC/FSC. FSC correlates with the cell volume while SSC correlates with a granularity of the cell. The areas from which cells were taken for subsequent analysis are shown. A total of 10,000–50,000 events were acquired for analysis; Figure S2: Western blot with N-protein antibodies for lysates of A431 cells stably transfected with plasmid coding for N-protein fused to the fluorescent protein mRuby3. The dotted line shows the area in which the intensities for the studied sample and for the background were determined. Samples shown: 1—A431 cells to which MNT was not added, 2—A431 cells that were incubated with 500 nM MHT_1_ for 15 h, 3—A431 cells that were incubated with 500 nM MHT_1_ for 24 h, 4—A431 cells that were incubated with 500 nM MHT_1_ for 39 h, 5—A431 cells that were incubated with 500 nM MHT_1_ for 48 h, 6—A431 cells that were incubated with 500 nM MHT_0_ for 48 h, M—marker of molecular mass in kDa; Figure S3: Western blot with N-protein antibodies for lysates of A549 cells stably transfected with plasmid coding for N-protein fused to the fluorescent protein mRuby3. Samples shown: 1—A549 cells to which MNT was not added, 2—A549 cells that were incubated with 500 nM MHT_1_ for 15 h, 3—A549 cells that were incubated with 500 nM MHT_1_ for 24 h, 4—A549 cells that were incubated with 500 nM MHT_1_ for 39 h, 5—A549 cells that were incubated with 500 nM MHT_1_ for 48 h, 6—A549 cells that were incubated with 500 nM MHT_0_ for 48 h, M—marker of molecular mass in kDa; Figure S4: Western blot with N-protein antibodies for lysates of A431 cells stably transfected with plasmid coding for N-protein fused to the fluorescent protein mRuby3. Samples shown: 1—A431 cells to which MNT_1_ and inhibitors were not added, 2—A431 cells that were incubated with 500 nM MHT_1_ for 24 h, 3—A431 cells that were incubated with 5 μM MG132 for 24 h, 4—A431 cells that were incubated with 500 nM MHT_1_ and 5 μM MG132 for 24 h, 5—A431 cells that were incubated with 100 nM Bafilomycin A1 for 24 h, 6—A431 cells that were incubated with 500 nM MHT_1_ and 100 nM Bafilomycin A1 for 24 h, M—marker of molecular mass in kDa.
