# Supplementary material for: Intracellular Degradation of SARS-CoV-2 N-Protein Caused by Modular Nanotransporters Containing Anti-N-Protein Monobody and a Sequence That Recruits the Keap1 E3 Ligase
Source: Pharmaceutics. 2023 Dec 19;16(1):4. doi: 10.3390/pharmaceutics16010004 (PMC10818351; doi:10.3390/pharmaceutics16010004)
Supplement: Supplementary file 1 [file pharmaceutics-16-00004-s001.zip › pharmaceutics-2722788-supplementary.pdf]

Supplementary Materials:

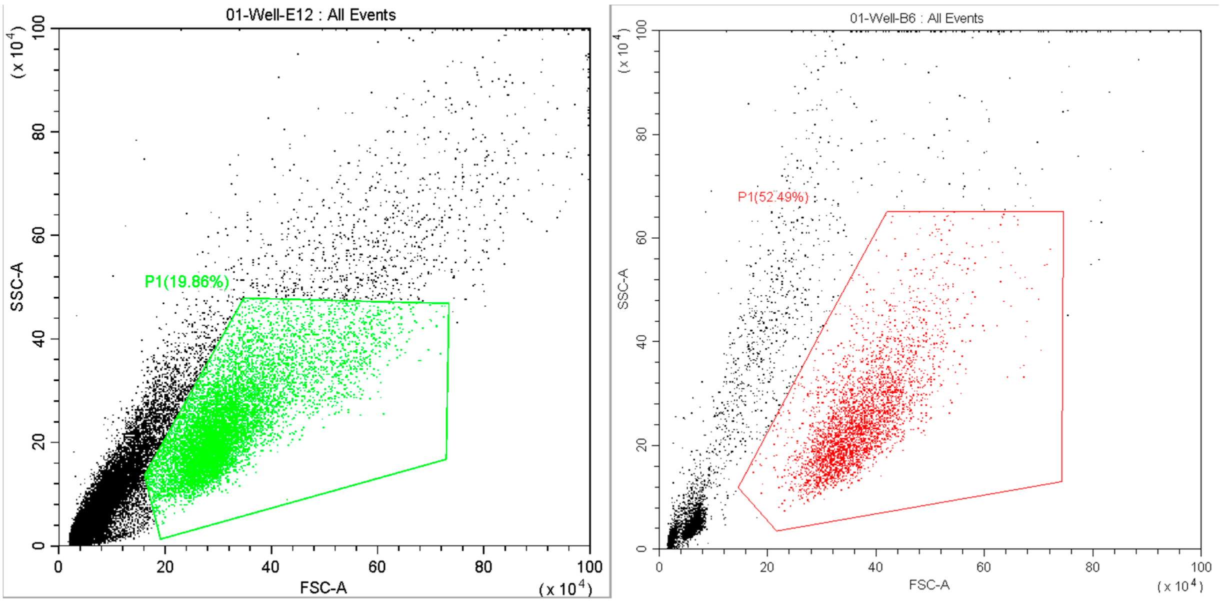

(a) (b)

Figure S1. Analysis of A431 (**a**) and A549 (**b**) cells stably transfected with plasmid coding for N-protein fused to the fluorescent protein mRuby3 using SSC/FSC. FSC correlates with the cell volume while SSC correlates with a granularity of the cell. The areas from which cells were taken for subsequent analysis are shown. A total of 10000–50000 events were acquired for analysis.

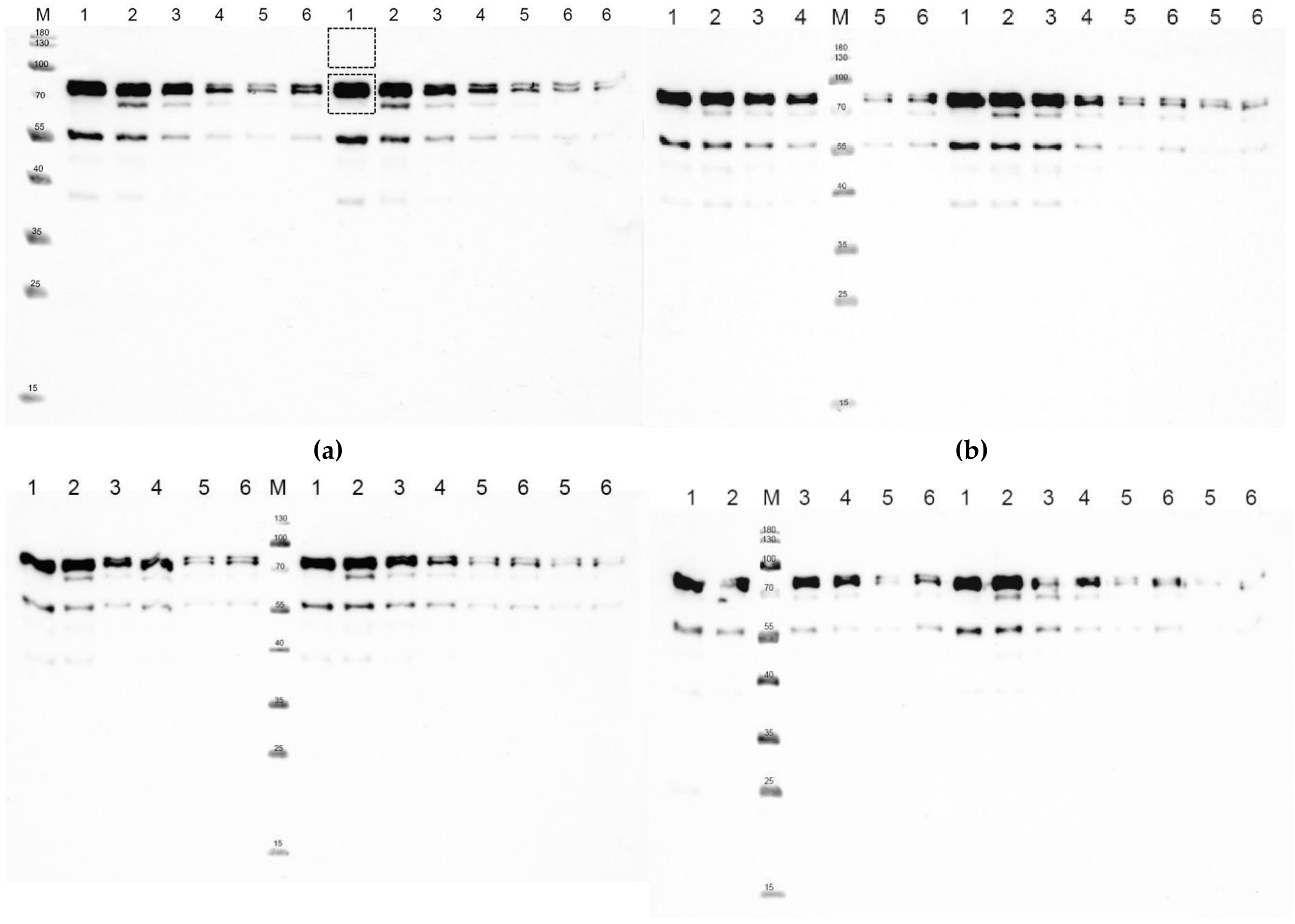

(a) (b) (c) (d)

Figure S2. Western blot with N-protein antibodies for lysates of A431 cells stably transfected with plasmid coding for N-protein fused to the fluorescent protein mRuby3. The dotted line shows the area in which the intensities for the studied sample and for the background were determined. Samples shown: 1—A431 cells to which MNT was not added, 2—A431 cells that were incubated with 500 nM MHT_1 for 15 hours, 3—A431 cells that were incubated with 500 nM MHT_1 for 24 hours, 4—A431 cells that were incubated with 500 nM MHT_1 for 39 hours, 5—A431 cells that were incubated with 500 nM MHT_1 for 48 hours, 6—A431 cells that were incubated with 500 nM MHT_0 for 48 hours, M—marker of molecular mass in kDa.

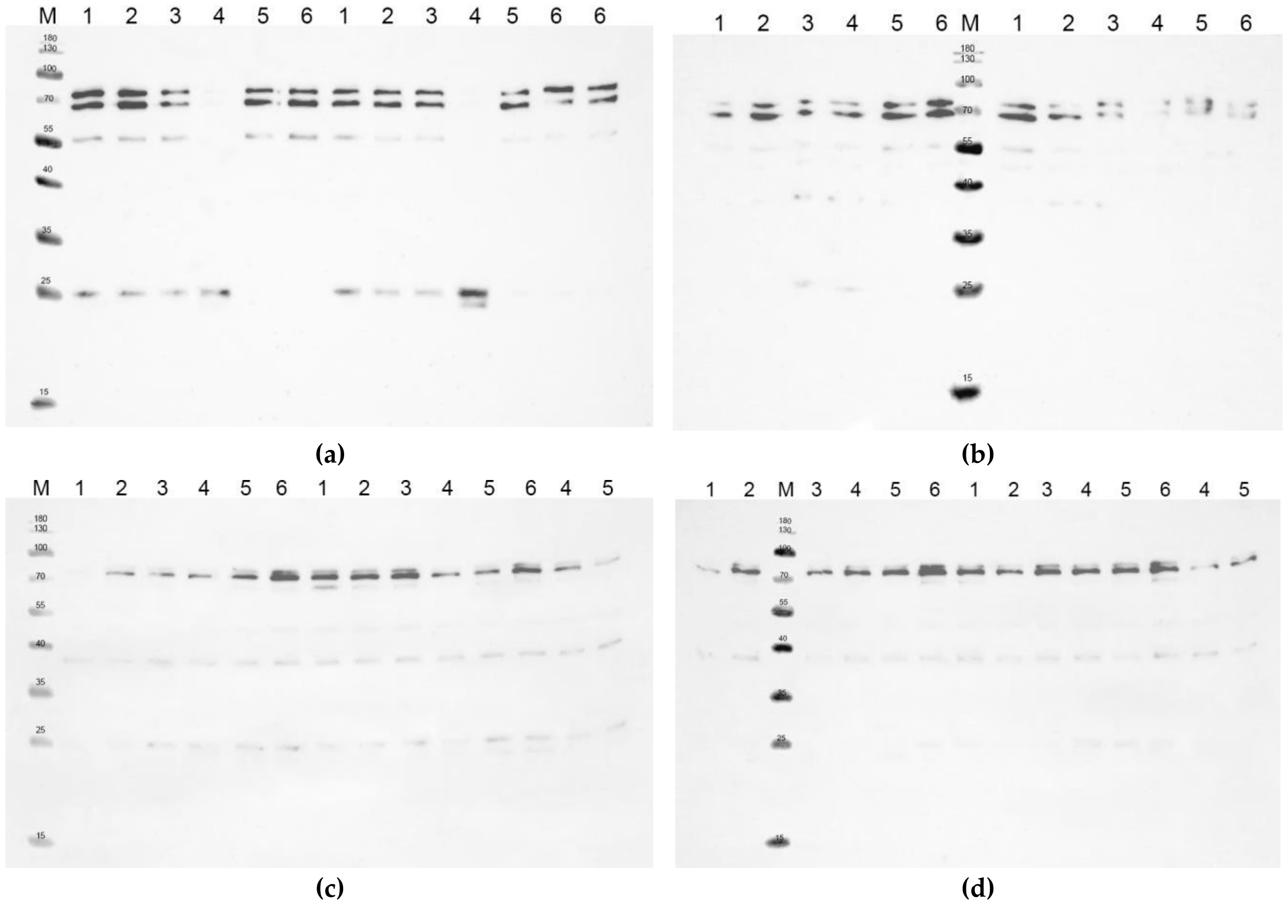

Figure S3. Western blot with N-protein antibodies for lysates of A549 cells stably transfected with plasmid coding for N-protein fused to the fluorescent protein mRuby3. Samples shown: 1—A549 cells to which MNT was not added, 2—A549 cells that were incubated with 500 nM MHT_1 for 15 hours, 3—A549 cells that were incubated with 500 nM MHT_1 for 24 hours, 4—A549 cells that were incubated with 500 nM MHT_1 for 39 hours, 5—A549 cells that were incubated with 500 nM MHT_1 for 48 hours, 6—A549 cells that were incubated with 500 nM MHT_0 for 48 hours, M—marker of molecular mass in kDa.

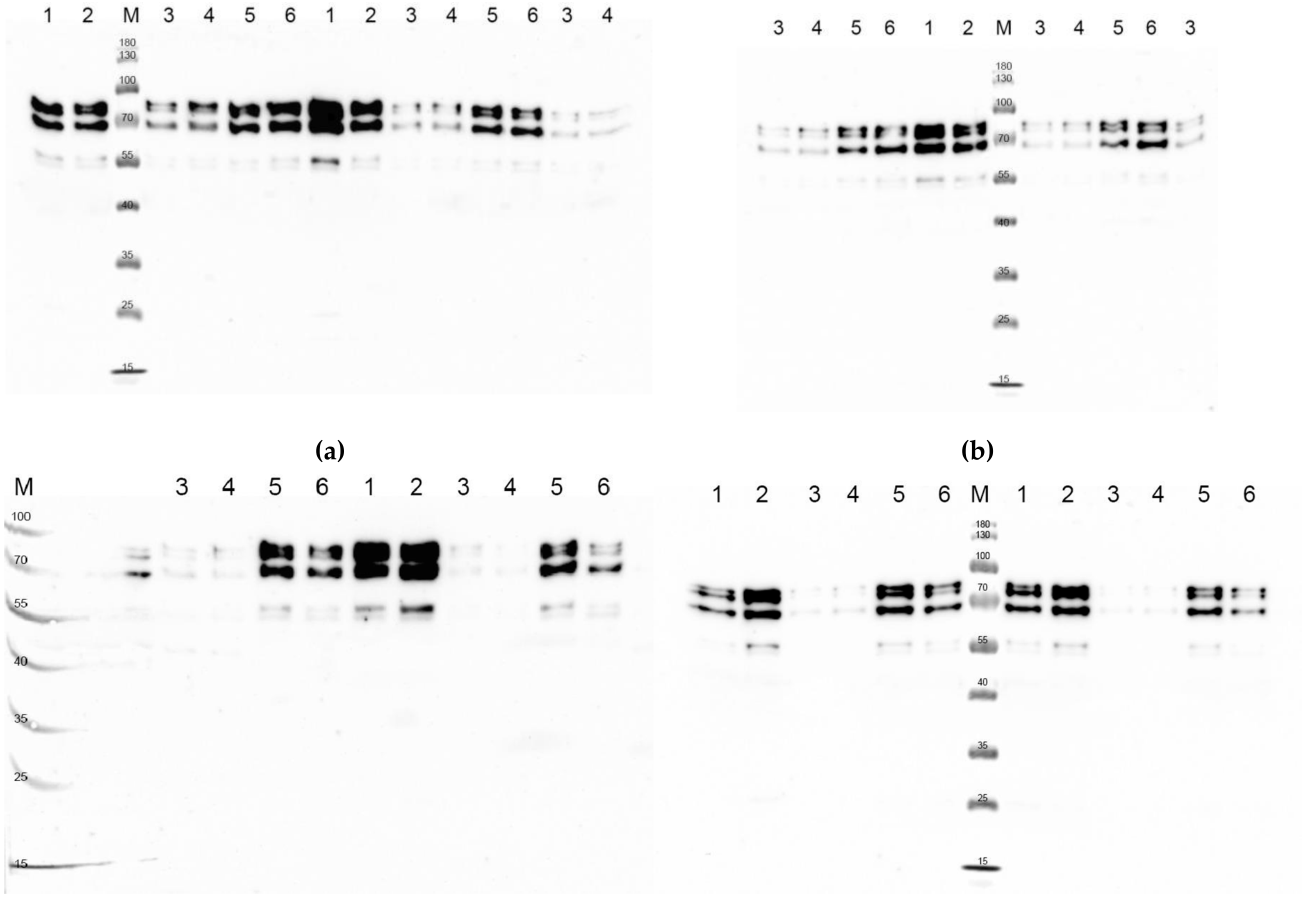

(a) **(b)** **(c)** **(d)**

Figure S4. Western blot with N-protein antibodies for lysates of A431 cells stably transfected with plasmid coding for N-protein fused to the fluorescent protein mRuby3. Samples shown: 1—A431 cells to which MNT_1 and inhibitors were not added, 2—A431 cells that were incubated with 500 nM MHT_1 for 24 hours, 3—A431 cells that were incubated with 5 μM MG132 for 24 hours, 4—A431 cells that were incubated with 500 nM MHT_1 and 5 μM MG132 for 24 hours, 5—A431 cells that were incubated with 100 nM Bafilomycin A1 for 24 hours, 6—A431 cells that were incubated with 500 nM MHT_1 and 100 nM Bafilomycin A1 for 24 hours, M—marker of molecular mass in kDa.
